# Androgen receptor decreases the cytotoxic effects of chemotherapeutic drugs in upper urinary tract urothelial carcinoma cells

**DOI:** 10.3892/ol.2013.1140

**Published:** 2013-01-18

**Authors:** TENG-FU HSIEH, CHI-CHENG CHEN, AI-LIN YU, WEN-LUNG MA, CAIXIA ZHANG, CHIH-RONG SHYR, CHAWNSHANG CHANG

**Affiliations:** 1 Division of Urology, Department of Surgery, Buddhist Tzu Chi General Hospital, Taichung Branch, Taichung 40427;; 2 Sex Hormone Research Center, China Medical University/Hospital, Taichung 40454, Taiwan, R.O.C.;; 3 George Whipple Lab for Cancer Research, Departments of Pathology, Urology and Radiation Oncology and The Wilmot Cancer Center, University of Rochester Medical Center, Rochester, NY 14642, USA

**Keywords:** androgen receptor, upper urinary tract urothelial carcinomas, chemotherapy, cytotoxic effect, chemoresistance

## Abstract

Upper urinary tract urothelial carcinomas (UUTUCs) represent relatively uncommon yet devastating tumors that affect more males than females. However, the correlation between gender difference and disease progression remains unclear. Androgen and the androgen receptor (AR) were previously hypothesized to account for the gender difference in the incidence of urothelial carcinomas; however, the role of AR in the development and progression of UUTUCs is not well understood. In addition, although UUTUCs are responsive to chemotherapy, various responses are presented among patients. Therefore, the aim of the present study was to determine the role of AR in the response of UUTUC cells to chemotherapeutic drugs. In this study, AR overexpression in UUTUC cells (BFTC 909) was identified to reduce the cytotoxic effect of chemotherapeutic drugs, including doxorubicin, cisplatin and mitomycin C and protected cells from drug-induced death. The expression of ABCG2, an ATP-binding cassette half-transporter associated with multidrug resistance, was increased in AR-overexpressing BFTC cells. In addition, use of the AR degradation enhancer, ASC-J9^®^, repressed the AR effect on increasing cell viability under drug treatment. In summary, results of the present study indicate that the status of AR expression levels in UUTUCs may be a significant factor in affecting the efficacy of chemotherapy and classic chemotherapeutic drugs and AR targeted therapy may provide a novel potential therapeutic approach to improve treatment of UUTUCs.

## Introduction

Upper urinary tract urothelial carcinomas (UUTUCs) are relatively uncommon tumors, accounting for ∼5% of all urothelial and 5–10% of all renal tumors. However, UUTUCs are associated with severe morbidity and mortality (1,2). These carcinomas are located more commonly in the renal pelvis than in the ureter, at a ratio of 3:1 (2,3). The incidence of bilateral UUTUCs is 2–8% (2,4). The development of UUTUC following primary diagnosis of bladder cancer is a rare event, occurring in only 2–4% of patients with bladder cancer (5); however, the development of secondary bladder cancer following primary UUTUC is more frequent with a risk of 20–50% (3,6–8). In addition, UUTUCs are more invasive and poorly differentiated compared with bladder cancer (9). Furthermore, microsatellite alterations in UUTUCs differ from bladder cancer (10). These observations indicate that UUTUCs and bladder cancer may exhibit specific variations with respect to cancer initiation and progression.

In a previous study, the 5-year disease-specific survival rates of the patients by primary tumor stage were identified as 100% for Ta/cis, 91.7% for T1, 72.6% for T2 and 40.5% for T3. Patients with primary stage T4 tumors had a median survival of 6 months (6). Radical nephroureterectomy with excision of an ipsilateral bladder cuff remains the gold standard for treatment of invasive UUTUCs (2,11,12). Since the development of metastatic disease leads to treatment failure in patients with locally advanced upper tract urothelial carcinoma and the high risk of disease relapse and cancer mortality for patients with stages III and IV UUTUCs, it is critical to prevent relapse following initial aggressive surgical therapy. Systemic adjuvant and neoadjuvant chemotherapy may prevent progression to metastatic disease and prolong survival (1,13). Furthermore, with increasing use of renal-sparing therapy by endoscopic means, chemotherapeutic agents are likely to become more important for the treatment of cancer, including urothelial carcinomas (14). Therefore, the development of a more effective adjuvant systemic therapy may provide significant benefit to patients with invasive UUTUCs.

UUTUC affects more males than females with a male-to-female ratio of 3:2 for tumors in the renal pelvis and 2:1 for those in a ureteral location (12). Disease-specific annual mortality is greater in females than in males (11). However, the mechanism by which gender difference affects the progression and prognosis is not clearly understood.

The role of androgen receptor (AR) in urothelial carcinoma of the ureter and renal pelvis remains unclear; however, AR has been demonstrated to affect urothelial carcinoma of the bladder (15). AR was identified in patients with localized and advanced transitional cell carcinoma of the bladder and kidney (16). AR protein expression has been found to decrease in tumors at higher pathological stages and grades, indicating that the loss of AR expression is associated with higher grade urothelial carcinomas (UCs) and invasive UCs, but has limited effect on the prognosis for survival (17,18). In a rat model of bladder carcinogenesis, testosterone was found to increase carcinogenesis (19). In addition, in a mouse model of bladder cancer and bladder cancer cells, androgens/AR were demonstrated to promote bladder cancer development and increase bladder cancer cell proliferation *in vitro* and xenograft tumor growth *in vivo*(15). Furthermore, in human transitional carcinoma AR-positive cell lines, knockdown of AR expression increased cell death and decreased proliferation and migration of bladder cancer cells. In xenograft models, AR knockdown in implanted bladder cancer cells suppressed AR-positive bladder tumor growth, indicating that AR is a potential therapeutic target for the treatment of bladder cancer (20). These observations indicate a role for AR in enhancing bladder cancer development. However, for other forms of urothelial carcinomas, the role of AR in UUTUC development and progression is not clear. We recently revealed that there is a positive correlation with higher AR expression found in superficial or low-grade UUTUCs of the ureter (21). However, the effect of AR on the therapeutic efficacy of chemotherapeutic drugs has not been previously investigated.

## Materials and methods

### Cell lines and chemicals

UUTUC cell line, BFTC 909 (from a UUTUC of a renal pelvis patient), was kindly provided by Dr Tzeng (Cheng Kung University, Tainan, Taiwan) and cultured in Dulbecco’s modified Eagle’s medium, containing 10% heat-inactivated fetal bovine serum (FBS) at 37°C in an atmosphere of 5% CO_2_(22).

To establish BFTC 909 cell lines overexpressing AR, cells were stably transfected with a pBabe-hAR plasmid using Lipofectamine (Invitrogen Life Technologies, Carlsbad, CA, USA) following the manufacturer’s instructions. Cells were selected using 1 *μ*g/ml puromycin and AR-overexpressing clones (as verified by western blot analysis) were named BFTC 909 pBabeAR1 and BFTC 909 pBabeAR2.

To exogenously express AR, a recombinant lentiviral vector containing wild-type AR (pWPI hAR) and a control lentiviral vector expressing the enhanced green fluorescent protein (pWPI) were used to overexpress AR. Lentiviral PWPI-AR/PWPI-control with pMD2.G packaging and psPAX2 envelope plasmids (lentivirus:packaging:envelopeZ, 2:1:1) were co-transfected into 293T cells. Following 48 h transfection, the target cells were cultured in the presence of viral supernatant containing 8 mg/ml polybrene (Millipore, Billerica, MA, USA) for 6 h. Flow cytometry was used to analyze cells overexpressing AR and dead cells were evaluated by propidium iodine (PI) under chemotherapeutic drug treatment.

Cisplatin (P4394) was purchased from Sigma-Aldrich (St. Louis, MO, USA). Doxorubicin hydrochloride and mitomycin C were obtained from the Hospital Pharmacy at China Medical University Hospital, Taiwan, China. ASC-J9^®^ (5-hydroxy-1,7-bis(3,4-dimethoxyphenyl)-1,4,6-heptatrien-3-one) was donated by AndroScience Corp. (San Diego, CA, USA).

### Cell viability assay

Viability of BFTC 909 cells was evaluated in 96-well plates 48 h after treatment by measuring cellular viability using the Cell Viability kit (XTT) (Roche Diagnostics, Indianapolis, IN, USA). The cytotoxic effects of chemotherapeutic drugs were determined by the colorimetric XTT assay based on the activities of mitochondrial enzymes in viable cells. Cells were seeded into 96-well culture plates at a seeding density of 1x10^4^ cells/well. After 24 h, complete medium was removed and changed to DMEM containing 10% FBS and cisplatin, doxorubicin or mitomycin C at designated concentrations. After 48 h, fresh DMEM containing 50 *μ*l XTT solution was added directly to the medium and cells were incubated for an additional 3 h at 37°C. A test wavelength between 450–500 nm and a reference wavelength of 650 nm were used in the assay.

### Protein analysis

For western blot analysis, protein extracts of each sample (100 *μ*g/lane) were electrophoretically separated and transferred onto nitrocellulose. Membranes were incubated with antibodies against AR or ABCG2 (Santa Cruz Biotechnology, Santa Cruz, CA, USA), followed by horseradish peroxidase-conjugated secondary antibody. Protein-antibody complexes were detected by an enhanced chemiluminescence system (Millipore, Bedford, MA, USA) using the Bio-Rad imaging system (Hercules, CA, USA).

### Quantitative real-time (qRT)-PCR

Total RNA was isolated using the TRIzol method (Invitrogen Life Technologies) according to the manufacturer’s instructions. Total RNA was reverse transcribed into cDNA using BluePrint RT Reagent Kit (Takara Bio, Inc., Shiga, Japan). The primer sequences were as follows: human AR, forward 5′-TGT CCA TCT TGT CGT CTT C-3′ and reverse 5′-CTC TCC TTC CTC CTG TAG-3′; human β-actin, forward 5′-TCA CCC ACA CTG TGC CCA TCT ACG A-3′ and reverse 5′-CAG CGG AAC CGC TCAT TGC CAA TGG-3′; and human ABCG2, forward 5′-GGG TTC TCT TCT TCC TGA CGA CC-3′ and reverse 5′-TGG TTG TGA GAT TGA CCA ACA GAC C-3′. qRT-PCR was performed using the Bio-Rad CFX96 real-time thermal cycler and SYBR Premix. Relative mRNA expression levels were normalized against β-actin (as an internal control) and determined by the 2^−ΔΔCt^ method.

### Cell death assay

PI exclusion was used to measure cell death. Cells were treated with various drugs for 48 h at 37°C. After 48 h, cells were trypsinized and resuspended in 1 ml PBS with 20 *μ*l PI (0.5 mg/ml) for 10 min at room temperature. Cell death was analyzed by flow cytometry (FACSCalibur; BD Biosciences, Franklin Lakes, NJ, USA).

### Statistical analysis

Statistical analysis was performed using Microsoft Excel using a two-sided Student’s t-test. Data are presented as mean ± SD. P<0.05 was considered to indicate a statistically significant difference.

## Results

### AR overexpression in BFTC 909 cells

**Figure 1:**
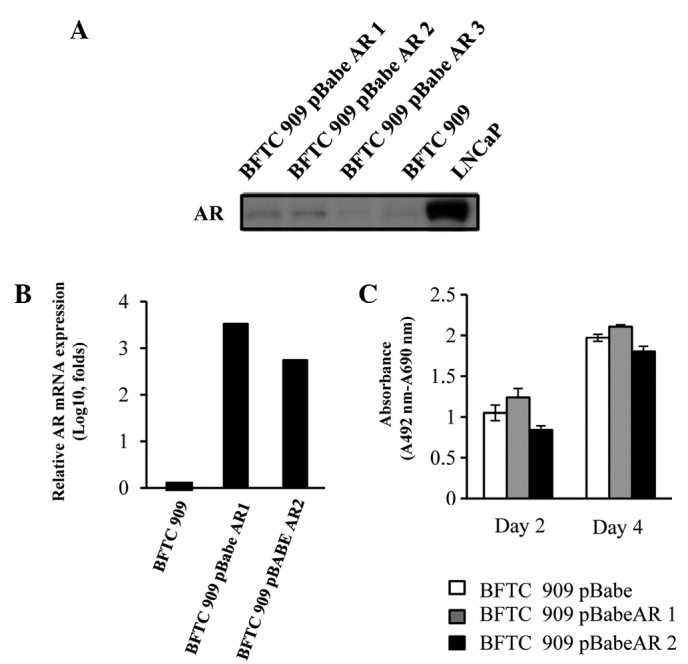
Detection of AR in UUTUC cells. (A) Western blot analysis of AR protein in BFTC 909 and BFTC 909 cells overexpressing AR. AR-positive human prostate cancer cells, LNCaP, were used as a control for AR expression. (B) Quantitative real-time PCR of AR mRNA expression in BFTC 909, BFTC 909 pBabeAR1 and BFTC 909 pBabeAR2. AR mRNA expression of BFTC 909 cells was set as 1. Results are expressed as log_10_ of the relative amount of AR mRNA normalized to β-actin. Data are representative of three independent experiments. (C) XTT assay analysis of BFTC 909 pBabe, BFTC 909 pBabeAR1 and BFTC 909 pBabeAR2 cell growth. Cells were cultured for 2 or 4 days. AR, androgen receptor; UUTUC, upper urinary tract urothelial carcinomas.

To determine the role of AR in the progression of UUTUCs, AR was overexpressed by stably transfecting AR cDNA into the UUTUC cell line, BFTC 909 (22). Two cell lines were generated (BFTC 909 pBabeAR1 and BFTC 909 pBabeAR2) by stable transfection. As demonstrated in Fig. 1, AR protein was increased in BFTC 909 pBabeAR1 and BFTC 909 pBabeAR2 and when comparing their relative AR mRNA expression with BFTC 909 control cells, the AR mRNA levels were increased 100-fold as compared with BFTC 909 control cells. In addition, expression of AR in BFTC cells did not affect cell growth (Fig. 1C).

### AR overexpression on viability of UUTUC cells treated with chemotherapeutic agents

**Figure 2:**
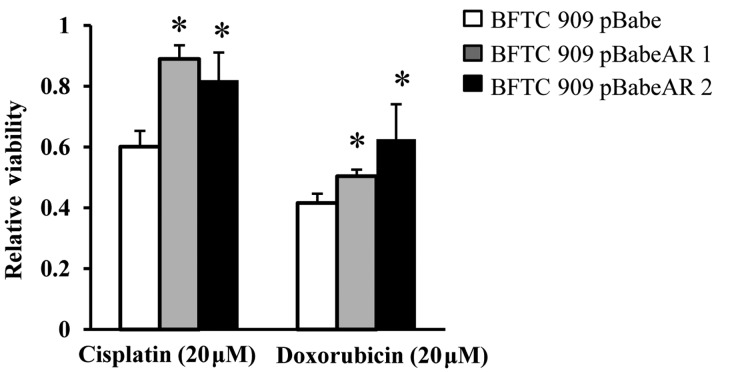
Cytotoxic effects of cisplatin and doxorubicin on BFTC 909 pBabe, BFTC 909 pBabeAR1 and BFTC 909 pBabeAR2 cells. Cells were treated with cisplatin and doxorubicin for 48 h and the cytotoxic effect was measured by XTT assay. Results are expressed as percentage of cell viability relative to no treatment control (viability was set at 1). All data are presented as the mean ± SD from at least three independent experiments. ^*^P<0.05, vs. control BFTC 909 pBabe cells.

To explore the effect of AR on cancer cells, the effect of AR on the cytotoxic effect of chemotherapeutic agents was determined. Adjuvant systemic chemotherapy is known to provide therapeutic benefit in patients and prevent recurrent bladder tumors with invasive UUTUCs (23,24). Cisplatin and doxorubicin have been used as anticancer drugs in the adjuvant chemotherapy of UUTUCs to improve the overall mortality rate associated with UUTUCs by targeting the remaining cancer cells (2,13). To investigate the potential role of AR during chemotherapy of UUTUCs, the effect of AR on the the cytotoxic effect of cisplatin and doxorubicin was analyzed. BFTC 909 pBabe, BFTC 909 pBabeAR1 and BFTC 909 pBabeAR2 cells were treated with cisplatin and doxorubicin for 2 days and the cytotoxic effect was measured by XTT assay to determine the viability. As revealed in Fig. 2, BFTC 909 pBabeAR1 and BFTC 909 pBabeAR2 cells exhibited higher viability compared with BFTC 909 cells stably transfected with vector control.

### Addition of AR via lentiviral vector on the viability and cell death of UUTUC cells treated with chemotherapeutic agents

**Figure 3:**
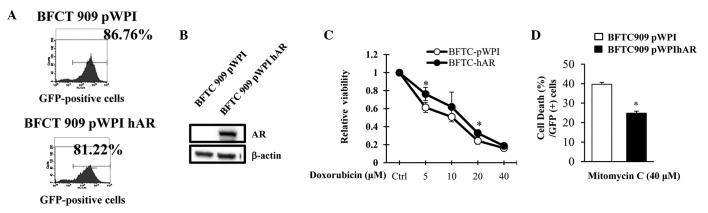
Overexpression of AR decreases the cytotoxic effect of doxorubincin and mitomycin C on BFTC 909 cells. (A) BFTC 909 cell infection with pWPI or pWPI hAR by lentiviral system revealed >80% infection efficiency as determined by GFP expression through flow cytometry. (B) AR protein was determined by western blot analysis. (C) Cells were treated with various concentrations of doxorubicin for 48 h and cellular viability was measured by XTT assay. Results are expressed as percentage of cell viability relative to no treatment control (viability set at 1). (D) Effect of AR on the mitomycin C-induced cell death was compared in BFTC 909 cells infected with pWPI or pWPI hAR by lentiviral system. Cells were treated with 40 *μ*M mitomycin C for 48 h. Cells were harvested and stained with propidium iodide and the dead cells analyzed by flow cytometry. All data are presented as the mean ± SD from at least three independent experiments. ^*^P<0.05, vs. BFTC-pWPI cells. AR, androgen receptor.

To further determine the effect of AR on UUTUC cells treated with chemotherapeutic drugs and reduce the potential artificial effects via overexpression AR using plasmids, BFTC 909 cells were transfected with a lentiviral system carrying pWPI hAR or control pWPI parental vectors expressing green fluorescent protein (GFP) and treated with doxorubicin (Fig. 3C). With similar infection efficiency (Fig. 3A), exogenous addition of AR via a lentiviral vector in BFTC cells (GFP-positive cells) increased the viability of cells under various concentrations of doxorubicin treatment compared with cells transfected with pWPI control vector. Mitomycin C is an additional chemotherapeutic drug used in UUTUC therapy (14,25). Therefore, the effect of AR status on cell resistance to mitomycin C was analyzed and mitomycin C was identified to induce more cell death in BFTC cells infected with lentiviral vector than in BTFC 909 infected with lentiviral vector expressing AR (Fig. 3D). These results demonstrate that the status of AR expression in BFTC cells plays a role in increasing the resistance to mitomycin C-induced cell death

### Expression of ABCG2 was increased in BFTC 909 cells over-expressing AR

**Figure 4:**
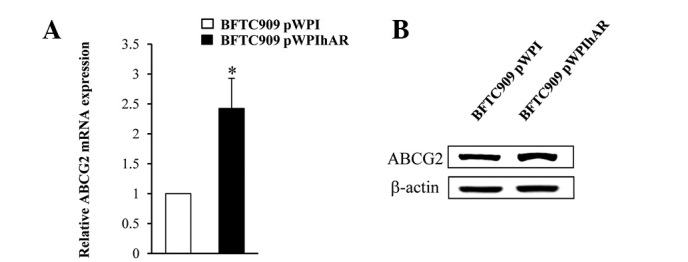
Expression of multidrug resistance gene, ABCG2, in BFTC cells with or without AR overexpression. (A) Real-time PCR analysis of mRNA expression of ABCG2 in BFTC pWPI and BFTC pWPI hAR cells. (B) ABCG2 protein expression in BFTC pWPI hAR and BFTC pWPI. Cell lysates were subjected to SDS-PAGE and antibodies against ABCG2. All data are presented as the mean ± SD from at least three independent experiments. ^*^P<0.05, vs. control cells. AR, androgen receptor.

The membrane transporter protein ABCG2 is linked to multi-drug chemoresistance in cancer cells due to its ability to reduce the intracellular concentrations of anticancer drugs using the energy of ATP hydrolysis to transport drugs across the cell membrane (26). To determine the mechanism by which AR overexpression by stable transfection or lentiviral infection results in increased viability of BFTC 909 cells, mRNA expression of ABCG2 was determined by qRT-PCR and protein expression of ABCG2 by western blot analysis. As demonstrated in Fig. 4A, BFTC pWPI-hAR cells expressed higher levels of ABCG2 compared with BFTC cells without AR overexpression. ABCG2 protein expression was also increased in BFTC pWPI-hAR cells compared with their counterpart controls (Fig. 4B).

### AR degradation enhancer, ASC-J9, blocked the effect of AR on the cytotoxity of chemotherapeutic drugs

**Figure 5:**
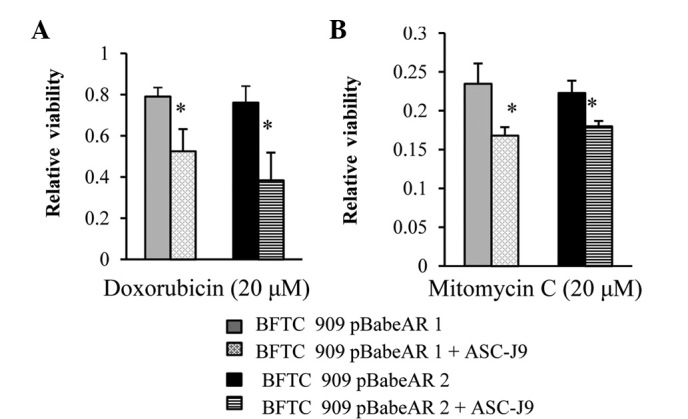
Effect of anti-androgen, ASC-J9, on the viability of BFTC 909 and BFTC 909 pBabeAR1 and pBabeAR2 cells treated with chemotherapeutic agents. BFTC 909 and BFTC 909 stable AR overexpressing cells were seeded in a 96-well plate and treated with (A) doxorubicin or (B) mitomycin C in the presence or absence of ASC-J9 for 48 h. Cellular viability was measured by XTT assay. All data are presented as the mean ± SD from at least three independent experiments. ^*^P<0.05, vs. control BFTC 909 pBabe cells. AR, androgen receptor.

Observations of the present study indicate that it may be possible to alter the response of UUTUC cells to the cytotoxic effect of chemotherapeutic drugs via alteration of AR expression in the cells to enhance their destruction. ASC-J9, the first AR degradation enhancer to be identified that degraded AR in selective cells with few side-effects (15,27–30) was applied to chemotherapeutic drug-treated cells to determine whether degradation of AR affects cell response to drugs. In the presence of ASC-J9, BFTC 909 cells overexpressing AR became more sensitive to doxorubicin treatment (Fig. 5A) or mitomycin C treatment (Fig. 5B).

## Discussion

Previous studies have hypothesized that gender is a factor in the incidence and progression of UUTUCs due to the observation that males have a substantially higher risk of developing UUTUCs. In addition, it has been identified that females tend to have more aggressive tumors (2,3,11). Although sex hormones and their receptors are involved in gender-specific differences, the exact roles of sex hormone receptors in UUTUCs remain unclear. AR has been previously demonstrated to promote urothelium carcinoma of bladder development (15,19), indicating that AR may be involved in UUTUC progression. In the present study, addition AR was revealed to lead to a decreased cytotoxic effect of chemotherapeutic agents, including cisplatin, doxorubicin and mitomycin C, on UUTUC cells, indicating that the status of AR expression affects the treatment and progression of UUTUCs during chemotherapy.

Although several studies examined the AR expression pattern in human bladder cancer, the prognostic significance of AR expression is inconsistent with studies demonstrating that loss of AR expression is associated with invasive bladder cancer (17,18) or has no association (31). Miyamoto *et al* demonstrated the role of androgen and AR in chemically induced mouse bladder cancer and identified that androgen and AR are involved in the carcinogenesis of bladder carcinoma, indicating that AR may be an androgen-independent carcinogenesis factor for bladder cancer development (15). It is likely that AR in the urothelium of the upper urinary tract may have a similar role in carcinogenesis. Accordingly, AR was proposed as a potential therapeutic target for the treatment of bladder cancer since the silencing of AR expression inhibits cell growth *in vitro* and *in vivo*(20).

Chemotherapy is used to increase lifespan and prevent disease recurrence following surgical resection of localized cancer. Chemotherapy is also utilized as part of the multimodal treatment of locally advanced cancer, allowing for more limited surgery with organ sparing and even cure. Therefore, more effective and less toxic chemotherapy regimens are likely to significantly benefit cancer patients. Cisplatin and doxorubicin have been previously used in adjuvant chemotherapy regimens for patients with upper tract transitional cell carcinoma and a positive outcome was reported (23,32). Mitomycin C was also applied in the chemotherapy of UUTUCs of the renal pelvis or ureter to prevent the recurrence following surgery with promising results (33,34). In the present study, BFTC 909 cells overexpressing AR were demonstrated to reduce the cytotoxicity of doxorubicin, cisplatin and mitomycin C in UUTUC cells, indicating that the presence of AR in BFTC 909 cells increases their drug resistance. These observations were confirmed further by expressing AR using a viral vector, revealing that following infection with pWPI hAR, BFTC 909 cells underwent less cell death with mitomycin C treatment than parent lentiviral vector-infected cells. Resistance to chemotherapy affects the drug efficacy and the factors affecting the chemoresistance of cells to chemotherapeutic drugs are largely associated with the multidrug-resistance-1 gene which encodes P-glycoprotein, whose function is to pump chemotherapeutic drugs from the inside of cells and from membranes to the outside (35). Other factors include drug inactivation, alterations in drug target, processing of drug-induced damage and evasion of apoptosis, all contributing to altered chemoresistance of cells (36). Whether AR affects these factors to alter chemoresistance requires further investigation. In the present study, however, the presence of AR was found to increase cancer cell chemoresistance and ABCG2 expression (Fig. 4). ABCG2 is an ABC transporter which belongs to a superfamily of transmembrane proteins that transport substrates across extra- and intracellular membranes and is associated with drug resistance (37). The increased level of ABCG2 in BFTC cells overexpressing AR may explain their higher viability under anti-cancer drug treatment.

Furthermore, the role of AR in promoting cancer is well characterized in prostate cancer with multiple mechanisms involved, including cell cycle regulation, apoptosis and kinase signals (38). However, the role of AR in UUTUCs is not well characterized. The results of the current study indicate that AR has the capability to increase the cell ability to resist cytotoxic agents and the mechanism may be associated with one of the multiple pathways of AR signaling. A previous study demonstrated that androgen, mediated by AR, induces Akt activation and causes the nuclear localization of a serine-threonine kinase, Akt/PKB (39). Akt/PKB is linked to cell proliferation, cell survival and anti-apoptotic pathways (40,41). It is possible that activation of Akt/PKB by AR may provide a survival advantage under the drug treatment.

To explore the clinical application of AR signaling on the treatment of UUTUC, ASC-J9, an AR degradation enhancer, which selectively targets AR without affecting libido, fertility and sexual behavior, was used. Our previous study demonstrated that ASC-J9 treatment led to degradation of AR in a number of cell lines and the effect was AR-specific. ASC-J9 was revealed to have therapeutic effects on spinal bulbar muscular atrophy mice via degradation of AR without affecting serum testosterone levels (30). ASC-J9 was also identified to improve wound healing (27). In addition, ASC-J9 was demonstrated to reduce AR-promoted tumor growth in liver (28) and bladder cancer (15). In the present study, ASC-J9 was combined with specific chemotherapeutic drugs to treat UUTUC cells, indicating that in AR-overexpressing cells, BFTC-pBabeAR1 and BFTC-pBabeAR2, ASC-J9 restored the cyototoxic effect of anti-cancer drugs, revealing that AR plays a significant role in the chemoresistance of UUTUC cells. In addition, the combination therapy of anti-AR and chemotherapeutic agents may exhibit an advantage for enhancement of the efficacy of chemotherapeutic agents in UUTUC cells with higher AR expression.

In the present study, AR was demonstrated to play a role in the suppression of the cytotoxic effect of doxorubicin, cisplatin and mitomycin C treatment to UUTUC cells, indicating that the AR expression status of UUTUCs affects chemotherapeutic efficacy. In addition, AR was identified as a novel therapeutic target in UUTUCs to increase the efficacy of chemotherapeutic agents. Therapies targeting the androgen-receptor signaling axis have been used for the treatment of prostate cancer with various strategies (42,43) and may be suitable for application in the treatment of UUTUCs. Thus, the combination of AR targeted therapy with chemotherapy may increase the efficacy of therapy to cure invasive and metastatic forms of cancer previously considered to be difficult to treat. Results of this study may aid understanding of the mechanism by which AR affects the progression of UUTUCs. More importantly, understanding the actions of AR in UUTUCs may lead to further understanding of the mechanisms of UUTUC progression and may identify novel targets for therapy.
